# Adverse drug reactions associated with amitriptyline — protocol for a systematic multiple-indication review and meta-analysis

**DOI:** 10.1186/s13643-020-01296-8

**Published:** 2020-03-17

**Authors:** Maria-Sophie Brueckle, Elizabeth T. Thomas, Svenja E. Seide, Maximilian Pilz, Ana Isabel Gonzalez-Gonzalez, Truc Sophia Nguyen, Sebastian Harder, Paul P. Glasziou, Ferdinand M. Gerlach, Christiane Muth

**Affiliations:** 1 Institute of General Practice, |Johann Wolfgang Goethe University, Theodor-Stern-Kai 7, D-60590 Frankfurt, Germany; 2 Faculty of Health Sciences and Medicine, Bond University, Gold Coast, Australia; 3 Institute of Medical Biometry and Informatics, University of Heidelberg, Heidelberg, Germany; 4 Goethe University, Institute of Clinical Pharmacology, Frankfurt, Germany

**Keywords:** Adverse drug reaction, Amitriptyline, Anticholinergic, Elderly, Multimedication, Multimorbidity, Multiple-indication review, Meta-analysis, Polypharmacy, Side effects

## Abstract

**Background:**

Unwanted anticholinergic effects are both underestimated and frequently overlooked. Failure to identify adverse drug reactions (ADRs) can lead to prescribing cascades and the unnecessary use of over-the-counter products. The objective of this systematic review and meta-analysis is to explore and quantify the frequency and severity of ADRs associated with amitriptyline vs. placebo in randomized controlled trials (RCTs) involving adults with any indication, as well as healthy individuals.

**Methods:**

A systematic search in six electronic databases, forward/backward searches, manual searches, and searches for Food and Drug Administration (FDA) and European Medicines Agency (EMA) approval studies, will be performed. Placebo-controlled RCTs evaluating amitriptyline in any dosage, regardless of indication and without restrictions on the time and language of publication, will be included, as will healthy individuals. Studies of topical amitriptyline, combination therapies, or including < 100 participants, will be excluded. Two investigators will screen the studies independently, assess methodological quality, and extract data on design, population, intervention, and outcomes ((non-)anticholinergic ADRs, e.g., symptoms, test results, and adverse drug events (ADEs) such as falls). The primary outcome will be the frequency of anticholinergic ADRs as a binary outcome (absolute number of patients with/without anticholinergic ADRs) in amitriptyline vs. placebo groups. Anticholinergic ADRs will be defined by an experienced clinical pharmacologist, based on literature and data from *Martindale: The Complete Drug Reference*. Secondary outcomes will be frequency and severity of (non-)anticholinergic ADRs and ADEs. The information will be synthesized in meta-analyses and narratives. We intend to assess heterogeneity using meta-regression (for indication, outcome, and time points) and *I*^2^ statistics. Binary outcomes will be expressed as odds ratios, and continuous outcomes as standardized mean differences. Effect measures will be provided using 95% confidence intervals. We plan sensitivity analyses to assess methodological quality, outcome reporting etc., and subgroup analyses on age, dosage, and duration of treatment.

**Discussion:**

We will quantify the frequency of anticholinergic and other ADRs/ADEs in adults taking amitriptyline for any indication by comparing rates for amitriptyline vs. placebo, hence, preventing bias from disease symptoms and nocebo effects. As no standardized instrument exists to measure it, our overall estimate of anticholinergic ADRs may have limitations.

**Systematic review registration:**

Submitted to PROSPERO; assignment is in progress.

## Background

About 50 million Europeans live with multimorbidity and the number is likely to continue rising [1]. Hence, general practitioners and many patients have to deal with the challenges of multimorbidity, and the multimedication that is frequently associated with it, on a daily basis [2]. Multimorbidity and multimedication are therefore a research priority in health care. The risk of drug-drug interactions in individual patients increases with the number of co-existing diseases and the number of (prescribed) medicines taken [3]. Depending on the individual patient’s conditions and on interactions between drugs and diseases, multimedication increases the risk of unwanted outcomes and serious harm [4]. It has been recommended that a distinction should be made between adverse drug reactions (ADRs) and adverse drug events (ADEs) [5]. ADRs are defined as “an *appreciable* harmful or unpleasant reaction, resulting from an intervention related to the use of a medicinal product; ADRs usually predict hazard from future administration and warrant prevention, or specific treatment, or alteration of the dosage regimen, or withdrawal of the product” [6]. ADEs are defined as “a *potentially* harmful effect resulting from an intervention related to the use of a medicinal product, which constitutes a hazard and may or may not be associated with a clinically appreciable adverse reaction and/or an abnormal laboratory test or clinical investigation, as a marker of an adverse reaction.” [5].

In the general population, the prescription of medicinal products with anticholinergic properties has risen by about 40% over the last 10 years [7]. Older age is known to increase the likelihood of being prescribed anticholinergic medicinal products because they are used to alleviate symptoms that often occur in later life, such as urinary incontinence, and sleep disorders [8]. As multimedication tends to increase anticholinergic burden in the elderly, the risk of ADRs is particularly high in medicinal products with anticholinergic effects. This is particularly true when a medicinal product with strong anticholinergic properties like amitriptyline is used, or when anticholinergic burden accumulates due to multiple prescriptions [9]. Another risk factor for anticholinergic prescriptions is institutionalization [10].

In 1961, amitriptyline was introduced to the US market as a tricyclic antidepressant (TCA), and it is still prescribed regularly for major depression [11]. It has been approved in more than 56 countries worldwide and is sold under the brand names Saroten, Elavil, and Endep, among many others [12]. In 2008, it was the third most frequently prescribed antidepressant in Germany, at 94 million defined daily doses (DDD) [13]. In addition to major depressive disorder, amitriptyline is used to treat other forms of depression, chronic pain, migraine, anxiety disorders [11], fibromyalgia [14], neuropathic pain [15], interstitial cystitis [16], nocturnal enuresis [17], eating disorders, and post-herpetic neuralgia [18].

Amitriptyline inhibits the reuptake of the neurotransmitters serotonin and norepinephrine into the presynaptic neuron by blocking their noradrenaline and serotonin transporters [19]. It also has an affinity for the muscarinic, histaminergic, and adrenergic systems. This affinity is considered significant for most of the effects of amitriptyline [19]. Nicotinic anticholinergic receptors (nAChR) are made up of 5 out of 17 subunits [20]. These receptors occur mostly on the cells of skeletal muscles, autonomous ganglia, and the CNS, and control neuromuscular transmission. Muscarinic anticholinergic receptors (mAChR) are divided into the subtypes M1-M5 and are mostly located in the central nervous system (CNS), heart, gastric tract, genitourinary tract, and respiratory tract. They control neuronal excitement, memory, learning, heart rate, analgesia, and extrapyramidal motor activity. Broadly speaking, ACh effects can be divided into periphery functions (control of skeletal muscles, autonomous nervous system, parasympathetic, enteral nervous system), and functions in the CNS (sleep-awake-rhythm, regulation, impulsion, vigilance, learning, memory) [20].

Commonly observed ADRs when taking anticholinergic medicines such as amitriptyline are constipation, dry mouth, dry eyes, tachycardia, urinary retention, agitation, confusion, delirium, falls, hallucinations, and cognitive dysfunction [21]. The following ADRs have been specifically reported for amitriptyline: vision problems, dizziness, sedation, sleepiness, somnolence, drowsiness, tremor, gastralgia, increased appetite, fatigue, asthenia, slow down, and sexual dysfunction. However, not all of these symptoms are caused by muscarinergic inhibition alone [11].

Anticholinergic ADRs are both underestimated and frequently overlooked in clinical management [21, 22]. Even though they are common, they are often regarded as “unavoidable” and as part of the aging process or disease course [23]. Failure to identify ADRs can lead to “prescribing cascades” [24, 25].This may result in the prescription of another medicinal product by the physician, or the increased use of over-the-counter products (OTC), rather than the discontinuation or dose adjustment of the responsible medicinal products [26]. With advancing age, an impeded ability to metabolize drugs increases the risk of impairment from anticholinergic burden [27, 28]. There is growing evidence to suggest that medicines with anticholinergic properties, such as amitriptyline, may cause serious physical and cognitive impairment in the elderly population [29]. Current research into amitriptyline concentrates mostly on its benefits and harms in relation to a single indication (e.g., depression [11]). We, in contrast, aim to investigate ADRs to amitriptyline across all indications. The objective of this systematic review and meta-analysis is thus to explore and quantify the frequency and severity of ADRs to amitriptyline vs. placebo in randomized controlled trials (RCTs) of adult humans across all indications, as well as in healthy individuals.

## Methods

This systematic review and meta-analysis will be conducted in accordance with the PRISMA statement [30].

### Aims

1. To assess ADRs caused by amitriptyline compared with placebo across indications, and in healthy subjects
2. To quantify their frequency
3. To quantify their severity
4. To quantify anticholinergic ADRs
5. To assess ADEs caused by amitriptyline compared with placebo across indications, and in healthy subjects
6. To identify factors that influence the frequency of ADRs and ADEs (e.g., age (mean per trial), dose, gender (proportion per trial), indication)
7. To examine the way ADRs and ADEs are described in the RCT under review (e.g., reporting individual reactions vs. reporting one overall number, active vs. passive assessment, subjective vs. objective assessment).

### Search strategy

We systematically searched multiple electronic databases including MEDLINE, Embase, PsycINFO, PsycLIT, Psyndex, and the Cochrane Central Register of Controlled Trials (CENTRAL) from inception. We combined free text searches with controlled terms such as Placebo AND (Amitriptyline OR Amitriptylines OR Amineurin OR Amitrip OR Amitriptylin OR Amitrol OR Anapsique OR Damilen OR Domical OR Elavil OR Endep) AND Randomized controlled trials (for the complete search strategy see Additional file 1).

We will perform citation analysis (forward and backward citation searches) on the studies included in Web of Science (including SCI - Science Citation Index Expanded, BIOSIS Citation Index, BIOSIS Previews, Current Contents Connect, Medline), and hand search the reference lists of systematic reviews. Furthermore, we will search the Food and Drug Administration (FDA) and the European Medicines Agency (EMA) databases for unpublished approval studies. Additionally, we will ask the major manufacturers of amitriptyline and other experts for information on further relevant RCTs.

We will search the clinical trial registries ClinicalTrials.gov, the International Standard Randomized Controlled Trial Number Register (ISRCTN), and the WHO International Clinical Trials Registry Platform (ICTRP) for unpublished studies.

### Study selection

Titles and abstracts of retrieved studies will be entered into Covidence© and independently screened for eligibility by two reviewers (MSB, ETT). Inclusion and exclusion criteria are provided in Table 1.

**Table 1:** Inclusion and exclusion criteria for study selection

PICOS framework	Inclusion criteria	Exclusion criteria
Population	Adult humans, age ≥ 18 any ethnicity, any sex, any indication, or healthy	Not applicable
Intervention	Amitriptyline monotherapy: any dose, frequency, duration, and oral mode of administration (tablets, capsules or liquid form)	Amitriptyline in combination with another active therapy, topical use of amitriptyline
Comparator	Passive placebo (an inert substance that may mimic the ADRs of amitriptyline, i.e., nocebo effects).	Comparing amitriptyline with active drugs or other forms of therapy
Outcome	Quantification of ADRs for each group, e.g., absolute numbers (frequency and severity (mean/sd)), associated with amitriptyline (i.e., negative effects): (a) caused by its anticholinergic properties and negative effects (b) not caused by anticholinergic properties	No outcomes relating to adverse drug reactions.
Study design	Randomized, double-blind, placebo-controlled trials (RCTs) with ≥ 100 subjects in total for the amitriptyline and placebo arm at baseline: no limitation on publication date, language, setting, time of follow up. Only the first period of crossover trials will be extracted (to avoid carry-over effects [31]. We will include trials with more than two arms, as long as we can identify an amitriptyline and a placebo arm.	Any other study design
Type of publication	Full publication	Abstract, conference presentation, provisional papers without complete results

Full texts will be obtained for all potentially eligible studies. The two reviewers will then independently determine eligibility based on inclusion and exclusion criteria. Previous to the assessment, a native speaker will translate full texts that are only available in languages other than English or German. Kappa statistics will be calculated to evaluate inter-observer agreement. Any disagreement over eligibility will be resolved through discussion with a third reviewer (CM). The detailed selection process will be shown in a PRISMA flow chart in the full review.

### Appraisal of methodological quality

The two review authors (MSB, ETT) will use the risk of bias (RoB) tool provided by Cochrane [32] to independently evaluate the risk of bias in eligible studies. Characteristics will be tabulated, and any disagreement between the two reviewers will be resolved by discussion with a third (CM and/or SES). The results of the RoB evaluation will be used in a sensitivity analysis.

### Data extraction

In order to carry out a pilot calibration exercise, we have selected 20 studies from a subsample of 40 studies included in the Cochrane Review “Amitriptyline versus placebo for major depressive disorder” [11]. We will develop and calibrate a standardized, electronic data extraction sheet and manual on the basis of the following data:

- Study metadata: authors, year, title, language, funding, country, setting, and objective (indication (Table 2), disease or symptom under review)
- Study design: type of randomization (e.g., individual or cluster-randomized), number of study arms, crossover, washout, type of blinding (single or double blinded)
- Population: age (mean per trial), gender (proportion per trial), sample size, baseline characteristics (including comorbidity and multimorbidity), completion rates
- Intervention: dose, frequency of dose delivery, days of treatment, and mode of application of amitriptyline, personalized dosage forms (e.g., maximum dosage), titration, intervention adherence, and concomitant medication
- Comparator: passive placebo
- Outcomes: type of ADR (e.g., anticholinergic symptoms (Table 3)) and other ADRs not commonly considered to be anticholinergic (Table 4), outcome reported subjectively (e.g., assessment scale) or measured objectively (e.g., measurement of salivation), type of data collection (e.g., systematically assessed or sporadically reported), time of measurement, length of follow-up, frequency of ADRs (number or proportion of subjects who have experienced an ADR), severity (e.g., mean, standard deviation) of ADRs, type and number of any reported ADEs (e.g., falls, fall-related fractures, deaths)

**Table 2:** Indications for amitriptyline

System	Indication
	Pain
Psychiatry	Depression, anxiety disorder, bulimia nervosa
Neurology	Depression with headache, headache, prophylaxis of migraine, prophylaxis of chronic tension-type headache, neuropathic pain, narcolepsy, pathological crying or laughing
Gastroenterology	Irritable bowel syndrome, interstitial cystitis
Rheumatology	Fibromyalgia
Toxicology	Medication-overuse headache, ciguatera poisoning

Source of data: *Martindale: The Complete Drug Reference* [33]

**Table 3:** Anticholinergic (antimuscarinic) adverse drug reactions to amitriptyline

System	Aggregated variables	Symptom
Gastrointestinal tract as	Dry mouth-related symptoms	Dry mouth, difficulty chewing, difficulty swallowing, difficulty speaking
	Digestion-related symptoms	Constipation
Genitourinary tract	Genitourinary-related symptoms	Urinary retention
Eye and adnexa	Vision-related symptoms	Blurred vision, disturbances in accommodation, and adaptation
Skin	Thermoregulation related symptoms	Hot dry skin, hyperthermia, anidrosis
Cardiovascular system	Cardiovascular-related symptoms	Tachycardia, palpitations, tachyarrhythmia
Nervous system	1. Fatigue-related symptoms	Fatigue
	2. Attention-related symptoms	Inability to concentrate, confusion, disorientation
	3. Memory-related symptoms	Mild amnesia, memory impairment
	4. Restlessness-related symptoms	Excitement, restlessness, agitation
	5. Coordination-related symptoms	Ataxia, hyperreflexia

Source of data: *Martindale: The Complete Drug Reference* [33], Collamati et al. 2016 [29]

**Table 4:** Non-anticholinergic adverse drug reactions to amitriptyline

System	Symptom
Gastrointestinal tract	Sour or metallic taste Stomatitis Gastric irritation Nausea and vomiting
Hypersensitivity reactions	Urticaria Angioedema Photosensitization Cholestatic jaundice Blood disorders such as eosinophilia Bone-marrow depression Thrombocytopenia Leucopenia Agranulocytosis
Endocrine system	Testicular enlargement Gynecomastia Breast enlargement Galactorrhoea Sexual dysfunction Changes in blood sugar concentrations Hyponatremia associated with inappropriate secretion of antidiuretic hormone
Other adverse effects	Increased appetite with weight gain (or occasional anorexia with weight loss) Sweating

Source of data: *Martindale: The Complete Drug Reference* [33]

To ensure data extraction is reliable, one reviewer will extract the data (MSB), and another will verify it (ETT). As we do not expect authors of studies published more than 10 years ago to respond to inquiries, we will only contact authors of studies published in 2009 or later in case of missing data, and then only when contact details (e-mail address) are provided in the publication. As research has shown that about 30% of trial authors are unreachable and 40% or more do not respond to emails, even after several reminders [34], we have decided that if no reply is forthcoming, or the message cannot be delivered, we will not try to contact the author again. The analysis will also include available data for studies published in 2008 or earlier.

### Outcomes

The primary outcome will be the frequency of anticholinergic ADRs as a binary outcome (absolute number of patients with/without any anticholinergic ADRs) in amitriptyline vs. placebo groups. For this purpose, we will separately retrieve the absolute number of anticholinergic ADRs (Table 3) for each type of reaction and for both treatment groups.

We generated the classification scheme by extracting ADRs in concern to the general population from *Martindale: The Complete Drug Reference* [33]and supplemented them with further reactions that are typical of an older population by using Collamati et al. [29]. The resulting list contained 39 items. In order to prioritize the symptoms on the list, an experienced clinical pharmacologist (SH) first rated them for specificity with regard to anticholinergic ADRs by differentiating between symptoms that are unequivocally caused by the inhibition of muscarinergic signaling [35, 36], and those that are not. Amitriptyline can also cause potassium channel blockades [37], peripheral alpha blockades [38], norepinephrine reuptake blockades [39], and inhibit histamine receptors [40], and sodium channels [41], all of which potentially cloud the clinical presentation of anticholinergic ADRs. The items on the list also needed to be distinguished from intoxication [42, 43]. Subsequently, a list of symptoms was drawn up, which then became the anticholinergic ADRs under study. The list consists of 26 ADRs in six organ systems. ADRs that were deemed to be sufficiently similar were pooled in groups of aggregated ADRs, such as vision-related symptoms (see Table 3).

Should the reported ADRs not allow the overall proportion of patients with/without anticholinergic symptoms to be calculated (e.g., when several symptoms occur in one patient, and ADRs are reported for each symptom), we will select the anticholinergic symptom that is most commonly reported. For example, if dry mouth is reported as the most frequent ADR in most of the studies, it will be selected as the primary outcome. Secondary outcomes will be the frequency and severity of any pre-specified anticholinergic and non-anticholinergic ADRs (Table 3 and Table 4). Additionally, we will look for any other ADRs and ADEs (e.g., falls and deaths) that are reported in the studies. For this purpose, we will separately retrieve the absolute number of ADRs/ADEs, as well as the group sizes, for each reaction and for both treatment groups. We will then classify the reactions according to the classification provided in Table 3 and Table 4.

### Data synthesis

Firstly, we will describe the data trial wise in terms of study characteristics such as study design, participants, dosing, harms detection, and others. Secondly, we will use forest plots to graphically display the frequency, nature, and severity of the ADRs, along with their confidence intervals, for each trial. We will provide a quantitative synthesis of findings from the included studies using the random-effects model with inverse variance weighting and the DerSimonian-Laird estimator to assess between-trial heterogeneity [44]. For each study, we will provide summaries of intervention effects by calculating odds ratios (OR) for dichotomous outcomes (such as the ADRs) and standardized mean differences for continuous outcomes (such as the severity of ADRs), along with their 95% confidence intervals. We do not expect to be able to measure time-to-event outcomes.

In order to assess publication bias, we will separately use funnel plots and the Egger regression test [45] for each outcome.

We expect the number of eligible studies to be high enough to permit us to perform a meta-analysis. However, as we expect trials to report a range of different outcome measures, we will assess comparability before conducting a quantitative synthesis.

### Statistics

Between-trial heterogeneity will be assessed by interpreting its magnitude relative to the scale on which the combined effect is reported, and by calculating the *I*^2^ statistic. When studies are sufficiently homogeneous, we will pool the results using a random-effects meta-analysis with standardized mean differences for continuous outcomes and odds ratios for binary outcomes, and we will calculate 95% confidence intervals and 2-sided *p* values for each outcome. We will use the DerSimonian-Laird estimator to assess between-trial heterogeneity [44]. Whenever more than five studies are included in the meta-analysis and the outcomes are binary, confidence intervals will be adjusted using the methods proposed by Hartung and Knapp [46–50]. Meta-regression will be used to explain heterogeneity at different time points, and for each indication and mode of administration. We will conduct sensitivity analyses based on study quality and the type of outcome reporting (objective vs. patient reported). We will use forest plots to illustrate the meta-analysis graphically, and will assess evidence of publication bias by means of funnel plots, and by assessing the skewness of the standardized deviates [51]. All analyses will be performed in R version 3.6.1 or higher [52].

#### Planned sensitivity and subgroup analyses

We will test the robustness of our results in sensitivity analyses that take into account the risk of bias as well as parameters of reporting quality.

For the subgroup analysis, we will look at mean age per trial. Provided enough studies are available, we will set the cutoff to ≥ 65 years for older/younger adults and ≥ 80 years for very old vs. younger and older adults. We will additionally perform subgroup analyses based on the proportion of males per trial, average duration of the treatment (≤ 1 week, ≥ 1 week, ≥ 4 weeks), average dosage (< 50 mg, 50–99 mg, 100–150 mg, > 150 mg), and indication. Assuming it is possible, we will also base the analyses on the proportion of frail patients per trial, proportion of disabled patients per trial and multimorbidity (≥ 3 diseases on average per trial vs. < 3 diseases on average per trial).

## Discussion

The results of this review will quantify the frequency of anticholinergic ADRs in adults taking amitriptyline compared with placebo for all indications and provide information on the most common adverse drug reactions and associated symptom patterns. Current evidence on anticholinergic effects is often based on observational data (e.g., [53, 54]), which may be biased, as they cannot distinguish anticholinergic symptoms from disease symptoms and nocebo effects [55]. In the meta-analysis of Ruxton et al. that studied the anticholinergic side effects of antidepressants in a total 124,286 participants, data were derived mostly from prospective or retrospective cohorts [54].

A major strength of our multiple-indication review of randomized placebo controlled trials is the design of our study. The first of these is the restriction to the studies with placebo group controls for disease-related symptoms and nocebo effects. Secondly, including studies of all conditions increase the power to detect rare ADRs, and small but important increases in common symptoms. RCTs are usually underpowered and the unintended effects are expected to be the same, regardless of the indication for the intervention [56–60]. Thirdly, our review avoids the problem of “overlapping” when a multiple-indication review consists of systematic reviews that include some, but not all, of the same studies [56]. A limitation is the clinical heterogeneity and specifically the potential interaction with the type of condition. We therefore pre-specified condition subgroups for analysis.

A major limitation is also that a single expert drew up the list of pre-specified anticholinergic ADRs rather than a group of interdisciplinary experts. Some subjectivity in the selection of symptoms summarized in our primary outcome, anticholinergic ADRs, is therefore possible. A further limitation is the restriction to RCTs with a sample size of 100 or more study participants. This will decrease the power of our analyses and limit our ability to detect rare events [57]. On the other hand, the combination of very small-scale studies with RCTs involving many participants would increase the heterogeneity between trials [56]. Other sources of heterogeneity, such as age of participants, indication, dose, duration of treatment, and methodological quality of studies, will be analyzed carefully. Another potential limitation is that our search and selection process may miss relevant studies.

As this is the first systematic review of randomized controlled trials of anticholinergic ADRs of amitriptyline across multiple indications, no direct comparison with existing literature is possible. However, we identified systematic reviews on amitriptyline that focused on single indications and included an assessment of harms [11, 14, 15, 61]. We will be able to compare our results with those published in these reviews, as some of our pre-specified ADRs overlap.

The results of our multiple-indication review will support clinical decision-making in adult patients and help physicians decide whether the expected benefits of amitriptyline will outweigh potential harms arising from anticholinergic ADRs.

## Supplementary information

**Additional file 1.** Complete search strategy, [Pdf document containing our complete search strategy]

## Data Availability

The datasets used and analyzed for the current study are available from the corresponding author on reasonable request after completion of the review.

## Abbreviations

Ach: Anticholinergic
ADE: Adverse drug event
ADR: Adverse drug reaction
CNS: Central nervous system
DDD: Defined daily doses
EMA: European Medicines Agency
FDA: Food and Drug Administration
ICTRP: WHO International Clinical Trials Registry Platform
ISRCTN: International Standard Randomized Controlled Trial Number Register
mAChR: Muscarinic anticholinergic receptors
nAChR: Nicotinic anticholinergic receptors
OTC: Over-the-counter products
RCT: Randomized controlled trial
RoB: Risk of bias
SD: Standard deviation
TCA: Tricyclic antidepressant

## References

[1] World Health Organization. Integrating care for people with multimorbidity: what does the evidence tell us? 2017 [Available from: http://www.euro.who.int/en/about-us/partners/observatory/news/news/2017/04/integrating-care-for-people-with-multimorbidity-what-does-the-evidence-tell-us]. Last access: 19th August 2019.

[2] Muth Christiane, Harder Sebastian, Uhlmann Lorenz, Rochon Justine, Fullerton Birgit, Güthlin Corina, Erler Antje, Beyer Martin, van den Akker Marjan, Perera Rafael, Knottnerus André, Valderas Jose M, Gerlach Ferdinand M, Haefeli Walter E (2016). Pilot study to test the feasibility of a trial design and complex intervention onPRIoritisingMUltimedication inMultimorbidity in general practices (PRIMUMpilot). BMJ Open.

[3] Marengoni A, Onder G (2015). Guidelines, polypharmacy, and drug-drug interactions in patients with multimorbidity. BMJ.

[4] World Health Organization. A systematic review of the prevalence and risk factors for adverse drug reactions in the elderly in the acute care setting 1969 [Available from: https://www.ncbi.nlm.nih.gov/pmc/articles/PMC4257024/]. Last access: 1st July 2019.

[5] Aronson JK (2013). Distinguishing hazards and harms, adverse drug effects and adverse drug reactions : implications for drug development, clinical trials, pharmacovigilance, biomarkers, and monitoring. Drug Saf.

[6] Aronson JK, Ferner RE (2005). Clarification of terminology in drug safety. Drug Saf.

[7] Lemmer B, DP US, Ludwig W-D, Klauber J (2017). Bronchospasmolytika und Antiasthmatika. Arzneiverordnungsreport 2017.

[8] Chew ML, Mulsant BH, Pollock BG, Lehman ME, Greenspan A, Mahmoud RA (2008). Anticholinergic activity of 107 medications commonly used by older adults. J Am Geriatr Soc.

[9] Tune L, Carr S, Hoag E, Cooper T (1992). Anticholinergic effects of drugs commonly prescribed for the elderly: potential means for assessing risk of delirium. Am J Psychiatry.

[10] Kersten H, Wyller TB (2014). Anticholinergic drug burden in older people’s brain - how well is it measured?. Basic Clin Pharmacol Toxicol.

[11] Leucht C, Huhn M, Leucht S. Amitriptyline versus placebo for major depressive disorder. Cochrane Database Syst Rev. 2012(2):CD009138. 10.1002/14651858.CD009138.pub2 PMC11299154 23235671

[12] European Medicines Agency (EMA). Assessment report : Saroten and associated names 2017 [Available from: https://www.ema.europa.eu/en/documents/referral/saroten-article-30-referral-assessment-report_en.pdf]. Last access: 20th January 2020.

[13] Lohse MJM-OB (2009). Psychopharmaka.

[14] Moore RA, Derry S, Aldington D, Cole P, Wiffen PJ. Amitriptyline for fibromyalgia in adults (review). Cochrane Database Syst Rev. 2015;(7):CD011824. 10.1002/14651858.CD011824 PMC6485478 35658166

[15] Moore RA, Derry S, Aldington D, Cole P, Wiffen PJ. Amitriptyline for neuropathic pain in adults (review). Cochrane Database Syst Rev. 2015;(7):CD008242. 10.1002/14651858.CD008242.pub3 PMC6447238 26146793

[16] Foster HE, Hanno PM, Nickel JC, Payne CK, Mayer RD, Burks DA (2010). Effect of amitriptyline on symptoms in treatment naive patients with interstitial cystitis/painful bladder syndrome. J Urol.

[17] Gelbe Liste. [Available from: https://www.gelbe-liste.de/wirkstoffe/Amitriptylin-hydrochlorid-75-mg-Tabletten-Zum-Einnehmen_cbf1717e-b734-4aff-9b9b-6f4abcda5024?scope=produkt_24921#ade.]. Last access: 15.01.2019.

[18] MedlinePlus. [Available from: https://medlineplus.gov/druginfo/meds/a682388.html#other-uses]. Last access: 28th January 2019.

[19] Guaiana G, Barbui C, Hotopf M. Amitriptyline versus other types of pharmacotherapy for depression (review). Cochrane Database Syst Rev. 2007(2):CD004186. 10.1002/14651858.CD004186 12804503

[20] Strobach D. Anticholinerge Arzneistoffe Erkennen, erklären, ersetzen. Pharmazeutische Zeitung. 2013;41. [Availible from: https://www.pharmazeutische-zeitung.de/ausgabe-412013/erkennen-erklaeren-ersetzen/]. Accessed 19 Feb 2020.

[21] Feinberg M (1993). The problems of anticholinergic adverse effects in older patients. Drugs Aging.

[22] Magin PJ, Morgan S, Tapley A, McCowan C, Parkinson L, Henderson KM, et al. Anticholinergic medicines in an older primary care population: a cross-sectional analysis of medicines’ levels of anticholinergic activity and clinical indications. J Clin Pharm Ther. 2016. 10.1111/jcpt.12413 27349795

[23] Tune LE (2001). Anticholinergic effects of medication in elderly patients. J Clin Psychiatry.

[24] Rochon PA, Gurwitz JH (1997). Optimising drug treatment for elderly people: the prescribing cascade. BMJ.

[25] Rochon PA, Gurwitz JH (2017). The prescribing cascade revisited. Lancet.

[26] Mintzer J, Burns A (2000). Anticholinergic side-effects of drugs in elderly people. J R Soc Med.

[27] Beers MH (1997). Explicit criteria for determining potentially inappropriate medication use by the elderly. An update. Arch Intern Med.

[28] Holt S, Schmiedl S, Thürmann PA (2010). Potenziell inadäquate Medikation für ältere Menschen: Die PRISCUS-Liste. Deutsches Ärzteblatt.

[29] Collamati A, Martone AM, Poscia A, Brandi V, Celi M, Marzetti E (2016). Anticholinergic drugs and negative outcomes in the older population: from biological plausibility to clinical evidence. Aging Clin Exp Res.

[30] Moher D, Liberati A, Tetzlaff J, Altman DG (2009). Preferred reporting items for systematic reviews and meta-analyses: the PRISMA statement. J Clin Epidemiol.

[31] Elbourne Diana R, Altman Douglas G, Higgins Julian PT, Curtin Francois, Worthington Helen V, Vail Andy (2002). Meta-analyses involving cross-over trials: methodological issues. International Journal of Epidemiology.

[32] Higgins JPT, Altman DG, Sterne JAC. Chapter 8: Assessing risk of bias in included studies. In: Higgins JPT, Green S (editors). Cochrane Handbook for Systematic Reviews of Interventions Version 5.1.0 (updated March 2011). The Cochrane Collaboration, 2011. [Available from: www.handbook.cochrane.org]. Accessed 28 Jan 2020.

[33] Martindale: the complete drug reference. Amitriptyline. Royal Pharmaceutical Society 2019 [Available from: https://about.medicinescomplete.com/publication/martindale-the-complete-drug-reference/]. Last access: 4th February 2019.

[34] Abell B, Glasziou P, Hoffmann T (2015). Reporting and replicating trials of exercise-based cardiac rehabilitation: do we know what the researchers actually did?. Circ Cardiovasc Qual Outcomes.

[35] Aaltonen L, Syvalahti E, Iisalo E, Peltomaki T (1985). Anticholinergic activity in the serum of patients receiving maintenance amitriptyline or doxepin therapy. Acta Pharmacol Toxicol (Copenh).

[36] Penttila J, Syvalahti E, Hinkka S, Kuusela T, Scheinin H (2001). The effects of amitriptyline, citalopram and reboxetine on autonomic nervous system. A randomised placebo-controlled study on healthy volunteers. Psychopharmacology.

[37] Li H, Shin SE, Seo MS, An JR, Ha KS, Han ET (2018). Inhibitory effect of the tricyclic antidepressant amitriptyline on voltage-dependent K(+) channels in rabbit coronary arterial smooth muscle cells. Clin Exp Pharmacol Physiol.

[38] Leighton HJ (1982). Quantitative assessment of the pre- and postsynaptic alpha adrenoceptor antagonist potency of amitriptyline. J Pharmacol Exp Ther.

[39] Kremer M, Yalcin I, Goumon Y, Wurtz X, Nexon L, Daniel D (2018). A dual noradrenergic mechanism for the relief of neuropathic allodynia by the antidepressant drugs duloxetine and amitriptyline. J Neurosci.

[40] Richelson E (1979). Tricyclic antidepressants and histamine H1 receptors. Mayo Clin Proc.

[41] Dokken K, Fairley P (2018). Sodium channel blocker toxicity [text]: StatPearls publishing.

[42] Thanacoody HK, Thomas SH (2005). Tricyclic antidepressant poisoning : cardiovascular toxicity. Toxicol Rev.

[43] Marshall JB, Forker AD (1982). Cardiovascular effects of tricyclic antidepressant drugs: therapeutic usage, overdose, and management of complications. Am Heart J.

[44] DerSimonian R, Laird N (1986). Meta-analysis in clinical trials. Control Clin Trials.

[45] Egger M, Davey Smith G, Schneider M, Minder C (1997). Bias in meta-analysis detected by a simple, graphical test. BMJ.

[46] Hartung J (1999). An alternative method for meta-analysis. Biom J.

[47] Hartung J, Knapp G (2001). On tests of the overall treatment effect in meta-analysis with normally distributed responses. Stat Med.

[48] Hartung Joachim, Knapp Guido (2001). A refined method for the meta-analysis of controlled clinical trials with binary outcome. Statistics in Medicine.

[49] Jonkman JN (2002). A simple confidence interval for meta-analysis. Stat Med.

[50] Sidik K, Jonkman JN (2003). On constructing confidence intervals for a standardized mean difference in meta-analysis. Commun Stat Simul Comput.

[51] Lin L, Chu H (2018). Quantifying publication bias in meta-analysis. Biometrics.

[52] R Core Team. R: a language and environment for statistical computing. R Foundation for Statistical Computing, Vienna, Austria 2019 [Available from: https://wwwR-projectorg/] Last access: 28th January 2020.

[53] Coupland CA, Dhiman P, Barton G, Morriss R, Arthur A, Sach T (2011). A study of the safety and harms of antidepressant drugs for older people: a cohort study using a large primary care database. Health Technol Assess.

[54] Ruxton K, Woodman RJ, Mangoni AA (2015). Drugs with anticholinergic effects and cognitive impairment, falls and all-cause mortality in older adults: a systematic review and meta-analysis. Br J Clin Pharmacol.

[55] Sanderson C, Hardy J, Spruyt O, Currow DC (2013). Placebo and nocebo effects in randomized controlled trials: the implications for research and practice. J Pain Symptom Manag.

[56] Chen Y-F, Hemming K, Chilton PJ, Gupta KK, Altman DG, Lilford RJ (2014). Scientific hypotheses can be tested by comparing the effects of one treatment over many diseases in a systematic review. J Clin Epidemiol.

[57] Vandenbroucke JP (2004). When are observational studies as credible as randomised trials?. Lancet.

[58] Hansen MP, Thorning S, Aronson JK, Beller EM, Glasziou PP, Hoffmann TC, Del Mar CB. Adverse events in patients taking macrolide antibiotics versus placebo for any indication. Cochrane Database Syst Rev. 2015. 10.1002/14651858.CD011825.pub2 PMC6353052 30656650

[59] Hansen MP, Scott AM, McCullough A, Thorning S, Aronson JK, Beller EM (2019). Adverse events in people taking macrolide antibiotics versus placebo for any indication. Cochrane Database Syst Rev.

[60] Gillies M, Ranakusuma A, Hoffmann T, Thorning S, McGuire T, Glasziou P (2015). Common harms from amoxicillin: a systematic review and meta-analysis of randomized placebo-controlled trials for any indication. CMAJ.

[61] Guaiana G, Barbui C, Hotopf M. Amitriptyline for depression. Cochrane Database Syst Rev. 2007;(3):CD004186. 10.1002/14651858.CD004186.pub2 PMC12286419 17636748

